# Autologous full-thickness RPE-choroid graft to treat high-risk drusenoid pigment epithelial detachment without CNV

**DOI:** 10.3205/oc000151

**Published:** 2020-04-22

**Authors:** Hedwig Sillen, Joke Ruys, Pieter-Paul Schauwvlieghe, Marc Veckeneer

**Affiliations:** 1 Department of Ophthalmology, Antwerp University Hospital, Edegem, Belgium; 2 Department of Ophthalmology, ZNA Middelheim Hospital, Antwerp, Belgium

**Keywords:** macular degeneration, retinal pigment epithelium, drusenoid pigment epithelial detachment, RPE-choroid graft

## Abstract

**Objective:** To report on the survival of a retinal pigment epithelium (RPE)-choroid graft translocated to treat a patient with drusenoid pigment epithelial detachment (DPED).

**Methods:** We describe a patient with bilateral high-risk DPED where one eye was treated with RPE-choroid translocation surgery and followed up for more than two years.

**Results:** The RPE-choroid graft surgery was straightforward and the fully perfused graft was able to support stable vision of 0.5 Snellen acuity for more than two years despite the development of a choroidal neovessel at the edge of the graft. The vision in the fellow eye dropped from 0.5 to 0.2 Snellen in the same period.

**Conclusion:** RPE-choroid translocation may slow the progression of DPED to atrophy but it can also transform dry age-related macular degeneration (AMD) into neovascular AMD.

## Introduction

Although the majority of neovascular age-related macular degeneration (AMD) patients can now be treated effectively with anti-VEGF injections, there is still no treatment available for dry AMD. 

Drusenoid pigment epithelial detachment (DPED) arises from the confluence of large areas of soft drusen [1].

It is well documented that the natural history of eyes containing DPED is characterized by a high rate of progression to geographic atrophy [1], [2], [3].

Recently, Balaratnasingam et al. [4] examined the lifecycle of large DPEDs based on SD-OCT features. Their results demonstrate that maximal DPED height and diameter are inversely correlated with final visual acuity (VA) and that the appearance of intraretinal hyperreflective foci preceded the collapse of a DPED. 

In agreement with our experience, Balaratnasingam et al. [4] found that visual function is usually still good while the PED volume is at its largest. Once the DPED collapses, accelerated atrophy usually ensues and macular function is irreversibly lost. 

If a treatment would be available, significant growth of the PED and the appearance of intraretinal hyperreflective foci in a DPED could be considered as a signal to prompt intervention. 

We describe a patient with a DPED on the verge of collapsing who was treated with an autologous full-thickness retinal pigment epithelium (RPE)-choroid graft. To our knowledge, this is the first report of a RPE-choroid graft translocation to treat DPED. 

## Case description

An eighty-year-old female patient came to our department in August 2015. Snellen VA was 0.5 in both eyes and she was pseudophakic. 

Funduscopic examination demonstrated a large well-circumscribed DPED in both eyes. There were no signs of an underlying choroidal neovascular membrane (CNV). Fluorescein angiography (FA) showed in early phase a faint hyperfluorescence of the PED in both eyes. There was an increased pooling during the late phase, but no late leakage. 

SD-OCT B-scan demonstrated a large PED in both eyes with moderate hyperreflectivity beneath the RPE band in correspondence with a DPED [5]. There were no signs of CNV on OCT-angiography (OCTA).

**Figure 1:**
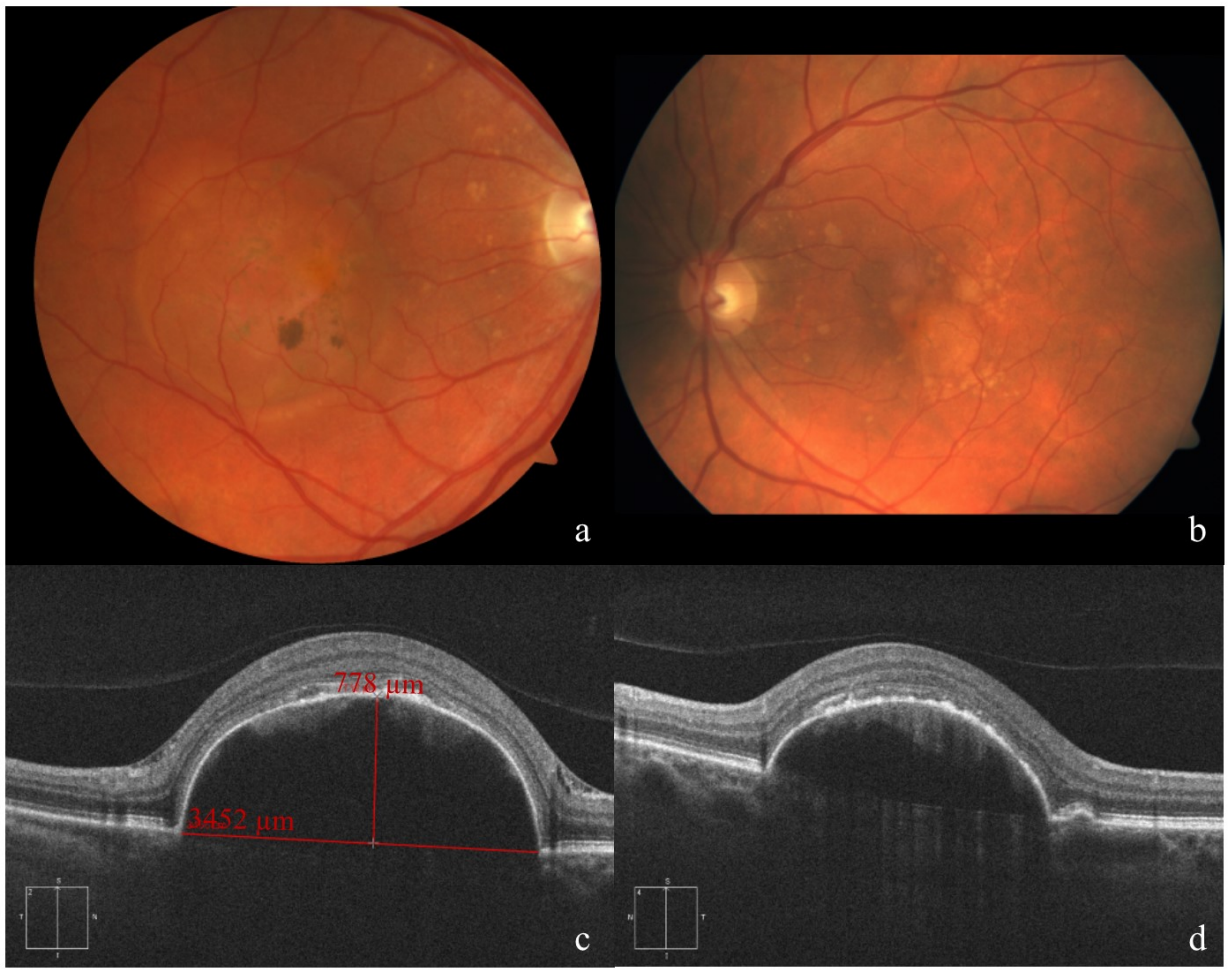
Fundus appearance and morphological evaluation of both eyes 4 weeks before operation of the right eye. Fundus photograph of the right (a) and left (b) eye, showing the drusenoid pigment epithelial detachment. Cross sections of SD-OCT images of the right eye (c), vertical scan with indications for height and diameter of the DPED, and the left eye (d).

On follow-up, the lesion size further increased. In December 2016, the DPED reached a diameter of 3,452 µm and a height of 778 µm (see Figure 1 (Fig. 1)). Inserting those values into the scatter plots used by Balaratnasingam et al. [4], we estimated the risk for DPED collapse and deterioration of function to be very high. In addition, the intraretinal hyperreflective material heralded possible imminent outer retinal atrophy. 

Since we have extensive experience with autologous RPE-choroid graft surgery to treat complicated neovascular AMD and DPED in dry AMD is thought to be the result of alterations in the RPE layer and Bruch’s membrane [2], [5], the possibility of translocation surgery was suggested as an option for the worst eye. After careful deliberation between the medical and surgical retina team and after extensive consenting of the patient, the surgery was planned. 

The right eye was operated in February 2017 by MV. The standard RPE-choroid graft procedure, as previously reported for neovascular AMD, was carried out under general anesthesia [6]. Of note, opening up the PED revealed a clear gelatinous substance without any signs of CNV. The material could not be grasped with forceps but could easily be removed with the vitreous cutter. The graft procedure was otherwise unremarkable. No problems were encountered during oil removal seven weeks later.

VA remained 0.5. Eight months after surgery SD-OCT images revealed a small subretinal fluffy bordered hyperreflective lesion with intraretinal cysts on the nasal border of the RPE-choroid graft, suggestive of a type 2 CNV, which was confirmed by FA. After treatment with intravitreal Aflibercept (Eylea), the lesion became sharply bordered with a few persisting intraretinal cysts on OCT. 

During further follow-up, the DPED in the left eye was found to develop similar characteristics predicting a collapse in the near future. Considering the CNV complication in the operated eye and the absence of long-term follow-up data of RPE-choroid graft for this indication, we decided to abstain from surgery for the left eye, despite insistence of the patient. 

**Figure 2:**
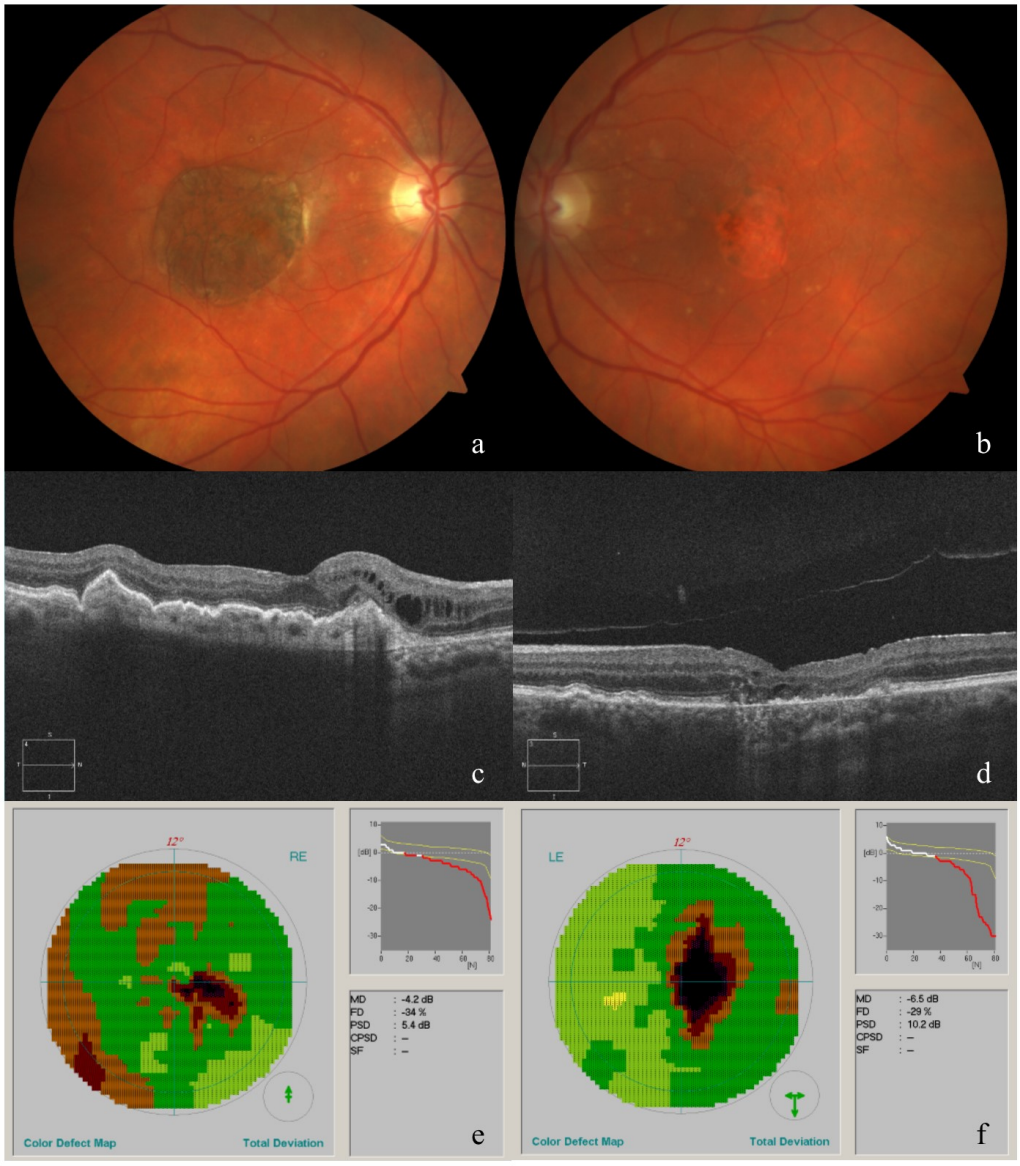
Fundus appearance and morphological and functional evaluation of both eyes at the last consultation. Fundus photograph of the right eye (a), showing the RPE-choroid graft, and left eye (b), showing macular atrophy. Horizontal cross sections of SD-OCT images of the right (c) and left (d) eye. Octopus visual field of the right (e) and left (f) eye.

At the last visit in July 2018, VA in the right eye was 0.6. The RPE-choroid transplant in the right eye appeared healthy on funduscopic examination and OCTA confirmed perfusion. The CNV on the nasal side of the graft showed no signs of activity. As expected for the left eye, the DPED collapsed and evolved into atrophy (see Figure 2 (Fig. 2)). VA in the left eye decreased to 0.35.

## Discussion

DPEDs are a frequent finding in dry AMD and their natural history is characterized by a high rate of progression to geographic atrophy [1]. The larger size of the DPED and the appearance of intraretinal hyperreflective lesions have been shown to be predictive of progression to atrophy [2], [7]. 

To date, no therapeutic options exist that can stabilize or improve VA in patients with DPED. 

We hypothesized that replacing the ‘diseased’ RPE-Bruch membrane-choriocapillaris complex by an RPE-choroid graft could rescue the overlying photoreceptors. 

It is important to keep in mind that RPE-choroid translocation surgery remains, in general, a very invasive treatment option that should only be considered in severe cases where other treatment options have failed or are non-existent. Even then, it is essential to weigh the potential benefits very carefully against the risk of postoperative complications including retinal detachment (with or without proliferative vitreoretinopathy), severe hemorrhage or graft failure. 

When considering RPE-choroid translocation surgery in cases of otherwise untreatable large DPEDs, timing is important. Surgery could be considered when collapse seems unavoidable. A PED diameter greater than 2 disc diameter and the appearance of intraretinal hyperreflective foci may be useful criteria. After all, when the DPED diameter was greater than 2 disc diameter, geographic atrophy occurred in 75% at long term as demonstrated by Roquet et al. [3]. Furthermore it is known that RPE activation and migration are important precursors to atrophy [8]. Another interesting biomarker for collapse is disruption of the RPE + basal lamina band [4], but we presume that damage to the photoreceptors is already present at that moment and a graft would not be justifiable anymore.

Another important issue to consider is the lifespan of the graft. Potential long-term survival is suggested by a recent report showing stable VA of 20/32 after 13.5 years, despite several complications [9], [10]. 

Taking into account the fact that the previously described high-risk characteristics of DPED were clearly present in our patient and were considered predictive of imminent atrophy while the outer retina still appeared healthy on OCT, we considered the timing for surgery to be optimal.

## Conclusion

We describe a patient who underwent RPE-choroid graft translocation surgery to treat a DPED on the verge of collapsing. Despite the postoperative development of a CNV at the edge of the graft requiring anti-VEGF injections, functional outcome was favorable compared to the natural history of the fellow eye during the one-and-a-half years of follow-up. 

Regarding the results of the RPE-choroid graft translocation surgery in this patient, a prospective, randomized trial appears necessary to evaluate safety and efficacy of such a procedure in DPED patients.

## Abbreviations

- RPE: retinal pigment epithelium
- PED: pigment epithelial detachment
- DPED: drusenoid pigment epithelial detachment
- AMD: age-related macular degeneration
- VEGF: vascular endothelial growth factor
- SD-OCT: spectral-domain optical coherence tomography
- VA: visual acuity
- CNV: choroidal neovascularization
- FA: fluorescein angiography
- OCTA: optical coherence tomography angiography

## Notes

### Competing interests

The authors declare that they have no competing interests.

### Informed consent

The authors declare that the patient‘s consent was received to use her medical records to write this case report.
